# Worry in imagery and verbal form: Effect on residual working memory capacity

**DOI:** 10.1016/j.brat.2010.11.005

**Published:** 2011-02

**Authors:** Eleanor Leigh, Colette R. Hirsch

**Affiliations:** a King’s College London, Institute of Psychiatry, UK; b University of Western Australia, Perth, Australia

**Keywords:** Worry, Working memory, Generalised anxiety disorder, Attentional control, Imagery

## Abstract

Worry-prone individuals have less residual working memory capacity during worry compared to low-worriers (Hayes, Hirsch, & Mathews, 2008). People typically worry in verbal form, and the present study investigated whether *verbal* worry depletes working memory capacity more than worry in imagery-based form. High and low-worriers performed a working memory task, random interval generation, whilst thinking about a worry in verbal or imagery form. High (but not low) worriers had less available working memory capacity when worrying in verbal compared to imagery-based form. The findings could not be accounted for by general attentional control, amount of negatively-valenced thought, or appraisals participants made about worry topics. The findings indicate that the *verbal* nature of worry is implicated in the depletion of working memory resources during worry among high-worriers, and point to the potential value of imagery-based techniques in cognitive-behavioural treatments for problematic worry.

Worry is characterized by repetitive intrusive negative thoughts about future events. Excessive and uncontrollable worry is a hallmark feature of generalised anxiety disorder (GAD), a disorder associated with chronic cognitive, social and occupational impairment (Kessler et al., 1994). High-worriers can be distinguished from low-worriers by perceived, and actual, uncontrollability of worrisome intrusions (Borkovec, Robinson, Pruzinsky, & DePree, 1983). We have previously suggested that basic cognitive processes may be implicated in this uncontrollability of problematic worry (Hirsch, Hayes, & Mathews, 2009).

Research has shown that anxiety is associated with reduced attentional control and working memory capacity (Derryberry & Reed, 2002; Eysenck, 1979; Eysenck & Calvo, 1992). According to the model proposed by Baddeley and Hitch (1974), working memory is a limited-capacity resource comprised of an attentional control system (Engle & Kane, 2004), referred to as the central executive; and two subsidiary systems (for verbal and visual information). Eysenck and Calvo (1992) suggest that worry is responsible for the detrimental effect of anxiety on working memory, and executive functioning in particular. These proposals have been supported in a number of experimental (Crowe, Matthews, & Walkenhorst, 2007; Derakshan & Eysenck, 1998; MacLeod & Donnellan, 1993; Rapee, 1993; Richards, French, Keogh, & Carter, 2000) and neuroimaging studies (Collette & Van der Linden, 2002; Santos, Wall, & Eysenck, submitted for publication). Rapee (1993) asked undergraduates to perform one of four tasks whilst simultaneously engaging in worry. The frequency of worry-related thoughts experienced during these phases was compared to the frequency reported when participants were engaging in worry without a second task. Two of the tasks assessed the central executive and either verbal (random generation of letters) or visuo-spatial processing (pressing buttons in a random pattern). The other two tasks tapped visuo-spatial (repeatedly pressing buttons in a particular pattern) or verbal (repeating the same letter) processing, but did not utilise the central executive. Only random generation of letters interfered with ability to worry, providing support for the proposal that worry is a verbal process that interferes with executive functioning. Hayes et al. (2008) directly assessed residual working memory capacity *during* worry. High (but not low) worriers showed more evidence of restricted working memory capacity during worry than when thinking about a positive topic. The authors concluded that depleted working memory resources during worry may be relevant to the persistence and perceived uncontrollability of worry. It may be that worry, once initiated, consumes working memory capacity. Inhibition of automatic responses and task switching (or ‘mental set shifting’) are both understood to be executive functions of working memory (Baddeley, 1966; Miyake et al., 2000). If worry depletes working memory, there may be less available working memory capacity to *inhibit* worrisome thoughts and *shift* to a more benign topic, maintaining the worry process (Hayes & Hirsch, 2007).

Worry is dominated by verbal thought with few images (Bergman & Craske, 2000; Freeston, Dugas, & Ladoucer, 1996) and people with clinical levels of worry tend to worry in verbal form more than non-clinical volunteers (Borkovec & Inz, 1990; Hirsch, Hayes, Mathews, Perman, & Borkovec, submitted for publication). Borkovec and colleagues (Borkovec, Alcaine & Behar, 2004; Sibrava & Borkovec, 2006) proposed that verbal worry serves a cognitive avoidance function in response to threatening and distressing information (such as images). In keeping with this are cognitive-behavioural approaches that encourage individuals to engage in coping imagery rather than verbal worry (Borkovec, Newman, Pincus, & Lytle, 2002). In line with the proposal that verbal worry functions to avoid distressing information such as images, Butler, Wells and Dewick (1992) found that participants asked to worry about a distressing film clip they had watched (of an accident at work) reported less anxiety immediately after the worry period compared to those who had been asked to think about it images. Importantly however, the individuals who had engaged in worry actually experienced more intrusive images about the film over the next few days than those who had engaged in imagery. This finding was replicated by Wells and Papageorgiou (1995). In a recent study by Stokes and Hirsch (2010) high-worriers were trained to engage in either verbal or imagery-based mentation about a personally relevant worry topic. Verbal worry was found to result in an increase in negative thought intrusions after the worry period. In contrast, imagery led to a decrease in negative intrusions. The authors conclude that the predominantly verbal nature of worry is implicated in the maintenance of worry.

The verbal mentation that characterizes worry, and particularly the worry of those with GAD, appears to maintain distressing intrusive thoughts and images, and therefore the worry process. Imagery, in contrast, does not seem to have this effect. One hypothesis is that verbally-based worry depletes available working memory capacity to a greater extent than worry in imagery-based form, leaving less resources available to inhibit or switch away from worrisome thoughts. Alternatively, it could be argued that high-worriers are ‘well-practised’ in verbal worry, and so might consume less capacity, as with expert performance acquired through extended practice (Ericsson & Kintsch, 1995). In this way verbal worry would be expected to deplete working memory to a lesser extent than worry in imagery, with no such effect among low-worriers.

The current study aimed to explore the effect of verbal worry on residual working memory capacity compared to imagery-based worry among people with high and low levels of worry. Consistent with the study of Hayes et al. (2008), we employed Baddeley’s (1966) dual-task method. Performance on a concurrent task (random generation) was used to measure residual working memory capacity whilst high and low-worriers engaged in verbal or imagery-based worry. Generating random sequences require high levels of attentional monitoring and control (Baddeley, 1986) to overcome the tendency to produce sequences that are well practiced (e.g. ascending or descending series). To the extent that limited working memory resources are consumed by other tasks (e.g. worry), the generated output is less random (Baddeley, Emslie, Kolodny, & Duncan, 1998).

Vandierendonck, De Vooght, and Van der Goten (1998) proposed a version of the random generation task in which participants tap a key at random intervals in time. Random interval generation (RIG) utilises central executive resources with minimal loading on verbal or visuo-spatial processes (Stuyven & Van der Goten, 1995; Vandierendonck, 2000; Vandierendonck, Kemps, Fastame, & Szmalec, 2004). Using the dual-task method, this RIG task allows evaluation of the comparable effect of verbal and imagery-based mentation on working memory. The current study developed and validated a method of analysing the data from this RIG task to derive widely used outcome measures (Towse & Neil, 1998).

In order to explore whether there were differences in performance on the RIG task when participants were not engaged in worry, high and low-worriers also completed the RIG task without a concurrent task. High-worriers report more negatively-valenced thought in general during worry compared to low-worriers, who typically report more neutral thoughts (Mathews & Milroy, 1994). They have also been found to appraise their worries more negatively than low-worriers (Vasey & Borkovec, 1992), and to endorse poorer attentional control in general than low-worriers (Pruzinsky & Borkovec, 1990). Participants were asked to rate thought valence during the experimental period, appraisals of their worries, and to complete a questionnaire designed to assess self-reported general attentional control, in order to rule out the possibility that these factors underpinned the hypothesized differences in working memory.

The aim of the study was to directly compare the effect of worry in imagery and verbal-linguistic form on residual working memory capacity among high and low-worriers. It was hypothesized that high-worriers would have less residual working memory capacity when engaged in verbal compared to imagery-based worry, which would not be accounted for by appraisals of worries, amount of negative content, nor self-reported general attentional control.

## Method

### Design

High and low-worry groups underwent a baseline RIG phase which comprised the single RIG task. This was followed by Imagery or Verbal Conditions which participants underwent in counterbalanced order (within groups). These comprised: a mentation training phase when participants received training in verbal thought or imagery; a worry focus phase, when participants focused on a worry in verbal thought or imagery; and a worry test phase when participants completed the RIG task whilst continuing to focus on their worry in the designated mentation style.

### Participants

Seventy-eight participants from King’s College London were selected based on scores on the Penn State Worry Questionnaire (PSWQ; Meyer, Miller, Metzger, & Borkovec, 1990). Those scoring above 55 were included in the high-worry group (Molina & Borkovec, 1994) and below 43 in the low-worry group (Gillis, Haaga, & Ford, 1995).

Seven participants were excluded as they reported that their thoughts about the worry topic (in either condition) were more than 50% positive. Twenty-three participants were excluded as they were not successful in worrying in the designated mentation type (less than 50% images in the imagery condition or words in the verbal condition, with no significant differences in the number of those excluded from the imagery or verbal condition, or the high and low-worry groups^1^). There were no significant differences between those included and excluded from the study in overall attentional control, as assessed by performance on single RIG task and scores on the ACS, nor with respect to age, gender, STAI-T, or PSWQ scores.

In the final sample, there were 24 participants in the low-worry group (PSWQ: *M* = 30.50, *SD* = 7.19), and 24 in the high-worry group (*M* = 63.46, *SD* = 5.18). Half the high-worry group and none of the low-worriers scored above cut-off on the Generalised Anxiety Disorder Questionnaire-IV (GAD-Q-IV; Newman et al., 2002), with a significant group difference (*χ*^2^(1) = 16.00, *p* < .01). The groups differed on the trait version of the State-Trait Anxiety Inventory (STAI-T; Spielberger, Gorsuch, & Lushene, 1970): low-worriers reported significantly less anxiety (*M* = 32.75, *SD* = 5.93) compared to high-worriers (*M* = 52.92, *SD* = 7.67; *t*(46) = 10.19, *p* < .01), and on the Attentional Control Scale (ACS; Derryberry & Reed, 2002), with low-worriers endorsing higher attentional control compared to high-worriers (*M* = 56.79 [*SD* = 8.12] vs. *M* = 45.13 [*SD* = 6.51]; *t*(46) = 5.47, *p* < .01). There were no significant gender differences between groups, with 20 female high-worriers and 14 female low-worriers (*χ*^2^(1) = 3.63, *ns*), nor were there significant age differences between high and low-worry groups (*M* = 25.57 [*SD* = 10.37] vs. *M* = 27.69 [*SD* = 10.90]; *t*(46) = 0.69, *ns*).

### Self-report questionnaires

The Penn State Worry Questionnaire (PSWQ; Meyer et al., 1990) consists of 16 statements about worry rated on a scale from 1 (*not at all typical of me*) to 5 (*very typical of me*), (total scores ranging: 16–80).

The Trait version of the State-Trait Anxiety Inventory (STAI-T; Spielberger et al., 1970) assesses how anxious individuals feel in general (scores ranging: 20–80).

The GAD-Q-IV (Newman et al., 2002) is a diagnostic instrument for GAD based on the Diagnostic and Statistical Manual-Fourth Edition (DSM-IV, 1994). The categorical scoring system was used in the present study (Newman et al., 2002) to indicate likely GAD diagnosis.

The Attentional Control Scale (ACS; Derryberry & Reed, 2002) is a 20-item measure of attentional ability (scores ranging: 20–80), with higher scores indicating better attentional control.

### Experimental task and training

#### Single RIG task

The RIG task (Vandierendonck et al., 1998) involves pressing the space bar on the keyboard approximately every second in a random and unpredictable rhythm. Participants had a 15-s practice trial and were given feedback on their performance, then the single RIG task for 5 min. The time interval between key presses was recorded in milliseconds.

#### Mentation style training

Participants were trained in verbal and imagery mentation (adapted from Stokes & Hirsch, 2010). To train participants to engage in imagery, they were asked to: “generate an image of the situation and tune in to what you can see, feel, smell, hear and taste in the image as though you are actually there right now”. Then in keeping with Holmes and Mathews (2005), participants were helped to imagine cutting a lemon. They undertook a further practice imagery exercise, in which they were asked to imagine cooking dinner. Participants gave feedback after each exercise on the extent to which they had been able to engage in imagery. If necessary, they were provided with further feedback on how to generate imagery. The training for participants in the verbal condition followed a similar procedure, except that participants were asked to think “in words, sentences and questions, as though you are talking to yourself”. Again, the experimenter gave the example of thinking about cutting a lemon in words and sentences. Participants were then asked to practice by thinking in verbal form about the topic of “friendship” for 1 min^2^.

#### Mood ratings

State mood was assessed a number of times. Anxiety and depression were rated on 100 mm visual analogue scales (VAS), ranging from ‘*not at all*’ to ‘*extremely*’. A measure of the change in anxiety over the worry test phase, termed ‘anxiety reactivity’ was calculated (based on these VAS ratings taken at various time points) for later analysis. This was done by first determining the change in anxiety over the test phase for each condition: [anxiety rating post-worry test phase – anxiety rating pre-worry test phase], then calculating the difference across the two conditions: [imagery anxiety reactivity– verbal anxiety reactivity]. The same was done for depression ratings.

#### Worry ratings

Worry topics were rated on three 100 mm VAS to measure how ‘Concerning’, ‘Personally Relevant’, and ‘Distressing’ they were, from ‘*not at all*’ to ‘*totally*’ to provide Worry Content Ratings.

For Worry Topic Ratings, an assessor rated the experimenter summary of worry topics (see Procedure below), in terms of negativity (Low; Moderate; High), and dominant topic (Social/Relationship; Physical; Work/Study; Financial). Inter-rater reliability analysis based on a randomly selected subset of 25% of participants showed good agreement, with full consensus for dominant topic ratings of both worry topics and for negativity for one worry topic (Cohen’s Kappa = 1.00, *p* < .01), and good agreement for the other worry topic (Cohen’s Kappa = 0.63, *p* < .05).

To provide Worry Appraisal Ratings, participants were asked to rate the worst outcome of their worry topics on three 100 mm VAS: “How likely is this to happen?”; “How catastrophic would it be?”; and “How well do you think you would cope with it?”, ranging from ‘*not at all*’ to ‘*totally*’.

#### Thought ratings

Participants rated the percentage of thoughts that were positive, negative and neutral during the worry test phases. Participants rated the extent to which they engaged in the designated mentation style during the Imagery and Verbal worry test phases, using two 100 mm VAS relating to amount of Images or Words, depending on condition, from ‘*not at all*’ to ‘*totally*’.

### Procedure

Participants were randomly allocated to Imagery-Verbal or Verbal-Imagery order within groups. Individuals completed the PSWQ, ACS, STAI and GAD-Q-IV. Participants identified two current worries and made Worry Content Ratings. The single RIG task followed. Participants rated their mood to assess baseline state anxiety and depression and then underwent verbal or imagery Mentation Training (depending on condition order). Participants were reminded of one of the identified worries and discussed salient aspects of it (noted down by the experimenter), and made Worry Appraisal Ratings. Participants focused on the worst outcome of the worry in either images or words (depending on condition) for 5 min (‘Worry Focus Phase’). They then continued to focus on the worry (in images or words) and carried out the RIG task simultaneously for a further 5 min (this was termed the ‘Worry Test Phase’). When the 5 min Worry Test Phase was completed, participants rated their current mood and then retrospectively rated their mood just prior to the Worry Test Phase (after the Worry Focus Phase). Participants then completed Thought Ratings in relation to the worry test phase. After the first condition, participants completed the Speed of Comprehension Test (Version A; Baddeley, Emslie, & Nimmo-Smith, 1992) as a filler task (Hayes et al., 2008). The procedure was repeated for the other condition, with participants first rating their mood to assess baseline state anxiety and depression.

### Analysis of RIG data

Randomness data was scored in the two ways considered the most sensitive (Baddeley et al., 1998; Towse, 1998) and in line with the suggestion of Towse (1998) to use more than one measure. Redundancy (*R*; Attneave, 1959; Baddeley, 1966) is a measure of the extent to which the same time intervals between key presses are made over the testing period as a whole (ranging from 0–100). Random Number Generation Score (RNG; Evans, 1978) reflects the extent to which *consecutive* intervals between key presses were the same in length (ranging 0–1). For both *R* and RNG scores, higher scores reflect *less* random performance, indexing less available working memory capacity. Towse and Neil (1998) provide a thorough explanation of these measures, and the relationship between them. *R* and RNG measures are calculated using RGCalc software (Towse & Neil, 1998), which analyses *discrete* data, for example numbers 1–10 on a random number generation task. We devised a system to transform data from the RIG task, which is *continuous*, to be analysed with RGCalc software^3^, as suggested by Towse and Neil (1998).

Each participant’s dataset was a series of time intervals (time difference between each pair of adjacent key presses), recorded using E-prime software (Schneider, Eschman, & Zuccolotto, 2002). The number of data points (time intervals) varied depending on how many key presses the participant made. To analyse the data, a discrete dataset was generated to represent this continuous variable of time intervals, i.e. time interval data was grouped into a discrete set of numbered groups (termed ‘bins’). First, the time interval data of *all* participants for all RIG phases (i.e. single RIG and both RIG Test Periods) was combined to create a variable made up of all time intervals for all participant data. This series of time intervals was reordered, from the shortest interval to the longest. Histograms and descriptive statistics were inspected to identify the range and distribution of the data. The aim was to group the responses on the basis of the length of the time interval, i.e. shorter time intervals were grouped with shorter time intervals, and longer intervals with longer ones. Based on the inspection of the reordered data, twenty interval bins were created each set at a width of 200 ms (i.e. first bin 0–200 ms; second bin 201 ms–400 ms etc.), except the final bin which was set at 3801 ms and above. Each participant’s original time difference interval data was then recoded into bins using this classification system, and this data was the discrete data analysed in RGCalc.

## Results

### Single RIG task

Two independent-samples *t*-tests were carried out on *R* and RNG scores comparing high and low-worriers’ performance on the single RIG task. Groups did not significantly differ for *R* scores (*t*(46) = 0.49, *ns*; *M* = 26.54 [*SD* = 10.45], *M* = 25.34 [*SD* = 10.70], respectively) or RNG scores (*t*(46) = 0.46, *ns*; *M* = 0.47 [*SD* = 0.13], *M* = 0.45 [*SD* = 0.14], respectively). This indicates that with no concurrent task demands, groups’ performance on the RIG task was equivalent.

### Randomness in imagery and verbal thought

A mixed MANOVA was carried out with *R* and RNG scores, comparing worry group (high vs. low), and the repeated measures factor condition (verbal vs. imagery). Mean randomness scores are shown in Table 1.

The main effect of condition was significant for both *R* (*F*(1, 46) = 12.18, *p* < .01, partial *η*^2^ = 0.21) and RNG scores (*F*(1, 46) = 7.70, *p* < .01, partial *η*^2^ = 0.14), with more random performance in the imagery compared to verbal condition (*R* scores: *M* = 27.38 [*SD* = 13.18] vs. *M* = 30.29 [*SD* = 14.67]; RNG scores: *M* = 0.46 [*SD* = 0.16] vs. *M* = 0.49 [*SD* = 0.17], respectively). There was no main effect of group for *R* (*F*(1, 46) = 2.49, *ns*) or RNG scores (*F*(1, 46) = 1.30, *ns*). Importantly, the interaction between condition and group was significant for both *R* (*F*(1, 46) = 9.30, *p* < .01, partial *η*^2^ = 0.17) and RNG scores (*F*(1, 46) = 7.59, *p* < .01, partial *η*^2^ = 0.14).

Pairwise comparisons showed high-worriers were less random in the verbal condition compared to imagery (*R*: *t*(46) = 4.62, *p* < .001; RNG: *t*(46) = 4.00, *p* < .001), with no difference in the low-worry group (*R*: *t*(46)<1, *ns*; RNG: *t*(46)<1, *ns*). High-worriers responded less randomly than low-worriers in the verbal condition although this was only significant for *R* scores (*R*: *t*(46) = 2.11, *p* < .05, *r* = 0.30; RNG: *t*(46) = 1.69, *ns*) with no group differences in the imagery condition, (*R*: *t*(46) = 1.07, *ns*; RNG: *t*(46)<1, *ns*).

A MANCOVA with group and condition as factors and anxiety reactivity as the covariate revealed the previously observed interaction between group and condition remained significant for *R* and RNG scores: *F*(1, 45) = 9.81, *p* < .01, partial *η*^2^ = 0.18; *F*(1, 45) = 8.58, *p* < .01, partial *η*^2^ = 0.16, respectively. This suggests that randomness differences are unlikely to be due to differences in changes in state anxiety. There was no significant main effect of anxiety reactivity or interactions with the covariate in the analysis with *R* or RNG scores (all *p* > .05). Analyses with depression reactivity as a covariate produced similar results.

Similarly, a MANCOVA with appraisals included as covariates revealed the interaction between group and condition remained significant for *R* and RNG scores: *F*(1, 43) = 8.85, *p* < .01, partial *η*^2^ = 0.17; *F*(1, 43) = 7.31, *p* < .01, partial *η*^2^ = 0.15, respectively. This suggests that randomness differences are unlikely to be due to appraisals made about the worry topics. There was no significant main effect of the appraisal covariates or interactions with the covariates in the analysis with *R* or RNG scores (all *p* > .05). For mean appraisal ratings, see Table 2.

A MANCOVA with group and condition as factors, and percentage of negative thoughts as a covariate showed the interaction between group and condition remained significant: *F*(1, 45) = 9.60, *p* < .01, partial *η*^2^ = 0.18; *F*(1, 45) = 7.89, *p* < .01, partial *η*^2^ = 0.15, for *R* and RNG scores respectively, suggesting that randomness differences are unlikely to be due to differences in reported negativity of the worry topic. There was no significant main effect of percentage of negative thoughts or interactions with the covariate in the analysis with *R* or RNG scores (all *p* > .05). Ratings of negative and neutral thought content during the worry test periods are shown in Table 3.

Similarly, a MANCOVA with ACS scores as the covariate revealed the interaction between group and condition remained significant for *R* and RNG scores: *F*(1, 45) = 8.11, *p* < .01, partial *η*^2^ = 0.15; *F*(1, 45) = 8.41, *p* < .01, partial *η*^2^ = 0.16, respectively, suggesting that randomness differences are unlikely to be due to differences in general attentional control. There was no significant main effect of ACS or interactions with the covariate in the analysis with *R* or RNG scores (all *p* > .05).

### Worry topic ratings

Analyses were conducted on assessor ratings and participants’ own ratings of the two identified worries to ensure they were equivalent. Paired *t*-tests comparing how currently concerning, distressing and personally relevant participants rated the two worries revealed no significant differences on any of these (for all: *t* < 1, *ns*).

Wilcoxon Signed Rank Tests on assessor ratings of descriptions of the two worry topics revealed no significant differences in terms of the negativity (*z* = −1.13, *ns*) or dominant topic (*z* = 0.55, *ns*).

### Mentation type in imagery and verbal conditions

Manipulation check data was investigated to ensure that participants engaged in the designated mentation style (imagery or verbal) to a similar extent during each condition. The mean rating of proportion of images reported by participants (on a VAS scale ranging from 0–100) during the imagery condition was 74.46 [*SD* = 13.36] for high-worriers and 73.89 [*SD* = 12.69] for low-worriers. The mean rating of proportion of words reported by participants (on another VAS scale ranging from 0–100) in the verbal condition was 76.58 [*SD* = 14.08] for high-worriers and 77.54 [*SD* = 16.97] for low-worriers.

An ANOVA was carried out to assess whether high and low-worriers were equally successful in engaging in imagery and verbal mentation styles. The ANOVA compared amount of mentation in the designated style (be that words or images) between group and condition. This revealed no significant main effect of group (*F* < 1, *ns*) or condition (*F*(1, 46) = 1.07, *ns*), or interaction (*F* < 1, *ns*). This suggests that high and low-worriers reported an equivalent amount of words in the verbal condition and images in the imagery condition.

### State mood at condition baseline and across the test periods

Mean mood ratings of state anxiety at condition baselines and before and after the worry test period are shown in Table 4. A mixed ANOVA was conducted comparing mood ratings (for anxiety ratings) across worry groups, conditions, and time (condition baseline, and before and after the worry test phase). This revealed a significant effect of group (*F*(1, 46) = 17.12, *p* < .01, partial *η*^2^ = 0.82), with high-worriers reporting more anxiety at all time points compared to low-worriers (*M* = 48.04 [*SD* = 26.00] vs. *M* = 26.62 [*SD* = 21.50]). There was a significant effect of time (*F*(2, 92) = 16.92, *p* < .01, partial *η*^2^ = 0.50). Pairwise comparisons indicated that state anxiety ratings before and after the worry test phase did not significantly differ (*p* = *ns*), but both differed significantly from anxiety rated at condition baseline (*p* < .01 for both). There was no significant effect of condition (*F*(1, 46) = 0.04, *p* = *ns*), or significant interaction between condition and group(*F*(1, 46) = 3.66, *p* = *ns*), time and group (*F*(1, 46) = 0.04, *p* = *ns*), condition and time(*F*(2, 92) = 2.67, *p* = *ns*), or condition, time and group (*F*(2, 92) = 0.52, *p* = *ns*).

## Discussion

For the first time, this study demonstrated that high-worriers have less residual working memory capacity when worrying in verbal form compared to imagery, whilst low-worriers do not differ across mentation type. In line with previous research, high-worriers showed poorer general attentional control (Derryberry & Reed, 2002), more negative thoughts during worry (Mathews & Milroy, 1994), more negative appraisals about worry topics (Vasey & Borkovec, 1992), and more anxious state mood compared to low-worriers (Borkovec et al., 1983). However, none of these factors accounted for the difference in working memory depletion across mentation type among high-worriers. Furthermore, high and low-worriers showed no difference in their performance on the RIG task when not engaged in worry.

Anxiety did not differ following worry in verbal or imagery form in either group. This is in contrast to some studies (Butler et al., 1992; Holmes & Mathews, 2005; Wells & Papageorgiou, 1995). One possibility is that the retrospective ratings of mood in the present study, used to minimise disruption of the worry process, may have been a less sensitive method of assessing state mood. However, increasingly findings are mixed regarding the impact of mentation style on self-reported mood; for example Merckelbach and colleagues (Merckelbach, Dijkstra, de Jong, & Muris, 1994) found no differences in subjectively or objectively recorded emotional reactivity when participants engaged in verbal or imagery-based processing. Neither Stokes and Hirsch (2010) nor Hirsch, Perman, Mathews, and Hayes (in preparation) found differences in state mood across imagery and verbal mentation. Both of these studies and the present study asked participants to focus on personal, current worry topics. In contrast, participants in the studies of Butler and colleagues (1992) and Wells and Papageorgiou (1995) engaged in worry or imagery about a novel film clip. Similarly, in the study of Holmes and Mathews (2005), participants were asked to focus on novel scenarios. It may be that mentation about a novel stimulus has a different effect on state mood compared to when the topic is personally relevant and familiar, as is the case with one’s own worries. Further research is needed to clarify the impact of mentation style on state mood.

Of note is the number of participants who were excluded for failing to engage in either/both mentation styles sufficiently. Importantly, approximately equal numbers were excluded from the two worry groups and from the two conditions, and there were no differences between those excluded and included on questionnaires or demographic characteristics. Further research could usefully develop the training procedures for mentation styles and it may be that enhanced training would lead to fewer participants being excluded. Given that individuals with problematic worry endorse an even greater predominance of verbal mentation in their worry (Hirsch et al., in preparation) enhancing training procedures for imagery mentation may be particularly important to consider for individuals with GAD. This will be valuable both in the context of further experimental studies and also for clinical interventions, such as those cognitive-behavioural interventions that incorporate imagery techniques (e.g. Borkovec et al., 2002).

The experimental manipulation was validated on the basis of participants’ ratings of the proportion of their worrisome thoughts that were in the designated mentation style. Of those participants included in the present study, ratings were comparable to those reported in a study comparing verbal thought and imagery among people with insomnia (Nelson & Harvey, 2002). In future studies it would be useful to include a measure of the proportion of worrisome thoughts in both mentation styles (i.e. words during the imagery condition and images during the verbal condition) to further validate the manipulation.

The study was carried out with a non-clinical sample and therefore caution must be taken in making generalisations about the findings. However, half the high-worriers were above the recommended cut-off on the GAD-Q-IV, indicating that they may meet criteria for GAD diagnosis. This suggests that this finding might also apply to a clinical population and it would be valuable and interesting to investigate the impact of verbal and imagery mentation on working memory capacity among individuals with GAD.

The present study has built on previous research indicating that reduced working memory capacity is associated with worry among high-worriers, with the finding that this is specifically dependent on the *type of mentation style*. This is in line with the recent finding of Stokes and Hirsch (2010) that high-worriers report more negative thought intrusions after a period of instructed verbal worry compared to imagery-based worry. But what is particularly unhelpful about the verbal mentation that characterizes typical worry and what is helpful about imagery? Self-report studies have found that verbal mentation tends to be less specific or concrete than imagery (Stöber, 1998), associated with “what if” type of thoughts. This lack of specificity is thought to impede problem-solving (Stöber, Tepperwien, & Staak, 2000) and emotional processing (Philippot, Baeyens, & Douilliez, 2006; Philippot, Schaefer, & Herbette, 2003), thereby maintaining the worry process. Watkins (2008) argued that abstract processing that characterizes repetitive negative verbal thought (including worry) perpetuates negative mood, in contrast to a more helpful concrete mode (such as that associated with imagery), which is also associated with more effective problem-solving and emotional processing (Philippot et al., 2003; Stöber, 1998; Watkins & Moulds, 2005).

The functions of working memory include inhibition and switching of attention (Baddeley, 1966; Miyake et al., 2000), and it may be that when engaged in typical (i.e. verbal) worry, high-worriers are then less able to shift away or stop worries, and also less able to problem-solve effectively. In contrast to verbal mentation, our findings indicate that when high-worriers *imagine* a worry topic, residual working memory resources are equivalent to that of low-worriers. We have suggested that working memory is required for inhibiting or shifting away from worrisome intrusions. In this way imagining a worry instead of verbally thinking about it may be helpful in interrupting the worry process. This provides tentative support for therapeutic interventions that incorporate imagery-based techniques (Borkovec et al., 2002).

A final and important question is why is the detrimental effect of verbal over imagery-based worry only found among high-worriers? People with high levels of anxiety or worry show cognitive biases towards threat (Hayes & Hirsch, 2007), making them more likely to detect threat and to interpret ambiguous stimuli in a threatening way. These biases require information storage and processing (Eysenck, Derakshan, Santos, & Calvo, 2007), which are functions of working memory (Engle & Kane, 2004). The working memory capacity of high-worriers is depleted by these information-processing biases compared to low-worriers, who show more benign biases (Hayes & Hirsch, 2007). In support of this, Hirsch et al. (2009) found that high-worriers who had undergone benign interpretation training subsequently showed greater residual working memory capacity whilst worrying compared to a control group of high-worriers. It may be that the relatively greater demand of verbal (compared to imagery-based) worry on working memory capacity is particularly unhelpful for high-worriers, because their resources are already depleted (by cognitive biases), leading to the persistence of worry.

In summary, the study has demonstrated that among high-worriers, verbally-based worry has a particularly detrimental effect on working memory resources compared to imagery mentation. Whilst future research is needed to explore whether this effect is present among a clinical population, the findings implicate verbal mentation in the persistence of problematic worry. In contrast, imagery-based mentation does not appear to deplete available working memory resources to the same extent. The findings add to the growing evidence base pointing to the potential benefits of engaging in imagery-based mentation for people with high levels of worry.

## Figures and Tables

**Table 1:** Mean *R* and RNG scores of High and Low-Worry Groups in the Imagery and Verbal Conditions (standard deviations in parentheses).

Worry group	*R* scores		RNG scores	
	Imagery condition	Verbal condition	Imagery condition	Verbal condition
High	29.15 (15.27)	34.62 (15.51)	0.47 (0.18)	0.53 (0.17)
Low	25.60 (10.71)	25.97 (12.66)	0.44 (0.14)	0.44 (0.17)

Note: *R* scores refer to redundancy scores; RNG scores refer to random number generation scores.

**Table 2:** Mean appraisals of worries identified for imagery and verbal conditions made by high and low-worry groups (standard deviations in parentheses).

Worry group	Imagery condition			Verbal condition		
	Appraisals			Appraisals		
	Catastrophic	Likelihood	Coping	Catastrophic	Likelihood	Coping
High	78.29 (20.31)	35.38 (19.36)	31.21 (31.49)	75.29 (18.84)	44.71 (22.02)	36.17 (28.83)
Low	55.71 (28.56)	37.42 (21.36)	57.38 (23.20)	47.75 (28.33)	42.83 (22.51)	59.67 (23.74)

**Table 3:** Percentage of negative and neutral worry content reported by high and low-worry groups in the imagery and verbal conditions (standard deviations in parentheses).

Worry group	Imagery condition		Verbal condition	
	Thought valence		Thought valence	
	Negative	Neutral	Negative	Neutral
High	71.96 (19.38)	16.25 (18.61)	69.33 (17.90)	13.89 (11.48)
Low	58.33 (26.73)	30.42 (24.40)	55.00 (23.82)	27.92 (23.50)

**Table 4:** State anxiety ratings reported by high and low-worry groups at condition baseline, and pre and post the worry test phases (standard deviations in parentheses).

Time point	High-worry group		Low-worry group	
	Imagery condition	Verbal condition	Imagery condition	Verbal condition
Condition baseline	34.71 (25.62)	42.50 (23.65)	17.03 (18.97)	15.50 (17.57)
Pre-worry test phase	55.75 (24.49)	53.33 (23.24)	36.42 (22.44)	31.63 (20.15)
Post-worry test phase	48.63 (26.69)	53.33 (28.21)	30.50 (22.01)	27.54 (23.02)

## Footnotes

1 Of those excluded from the High-Worry Group, nine were excluded on the basis of difficulty engaging in imagery-based mentation, and three on the basis of verbal mentation. Four low-worriers were excluded due to difficulties engaging in imagery, and seven on the basis of verbal mentation. Fisher’s Exact Tests did not indicate significant differences in the number of those excluded from the imagery or verbal condition, or the high and low-worry groups.

2 Different examples were used for the imagery and verbal mentation style training. On piloting it was found that imagery-based mentation of ‘friendship’ did not facilitate learning that processing style but imagery-based mentation of ‘preparing dinner’ did. In contrast, verbally thinking about ‘preparing dinner’ did not facilitate that processing style, whereas verbal mentation of ‘friendship’ did.

3 Piloting on real and simulated data was carried out to validate this method, and indicated it was valid. For details please contact corresponding author.
